# Short-Term and Long-Term Risk of Diabetes Mellitus among Patients with Spinal Cord Injury: A Nationwide Retrospective Cohort Study

**DOI:** 10.3390/healthcare12181859

**Published:** 2024-09-15

**Authors:** Seonghye Kim, Kyung-Do Han, Bongseong Kim, Ju-Hong Min, Won Hyuk Chang, In Young Cho, Dong Wook Shin

**Affiliations:** 1 International Healthcare Center, Samsung Medical Center, Sungkyunkwan University School of Medicine, Seoul 06351, Republic of Korea; 2 Department of Statistics and Actuarial Science, Soongsil University, Seoul 06978, Republic of Korea; 3 Department of Neurology, Samsung Medical Center, Sungkyunkwan University School of Medicine, Seoul 06351, Republic of Korea; 4 Neuroscience Center, Samsung Medical Center, Seoul 06351, Republic of Korea; 5 Department of Health Sciences and Technology, Samsung Advanced Institute for Health Sciences & Technology (SAIHST), Sungkyunkwan University, Seoul 03063, Republic of Korea; 6 Department of Physical and Rehabilitation Medicine, Center for Prevention and Rehabilitation, Heart Vascular Stroke Institute, Samsung Medical Center, Sungkyunkwan University School of Medicine, Seoul 06351, Republic of Korea; 7 Department of Family Medicine & Supportive Care Center, Samsung Medical Center, Sungkyunkwan University School of Medicine, Seoul 06351, Republic of Korea; 8 Department of Clinical Research Design and Evaluation, Samsung Advanced Institute of Health Science and Technology (SAIHST), Sungkyunkwan University, Seoul 03063, Republic of Korea; 9 Department of Digital Health, Samsung Advanced Institute of Health Science and Technology (SAIHST), Sungkyunkwan University, Seoul 03063, Republic of Korea

**Keywords:** spinal cord injury, diabetes mellitus, short-term, long-term, incidence, disability, level of injury

## Abstract

Objectives: Estimating the risk of diabetes mellitus (DM) is important for the proper management of patients with spinal cord injury (SCI). We investigated the short-term and long-term risks of DM among patients with SCI, according to the presence or severity of post-SCI disability and the level of injury. Methods: We conducted a retrospective cohort study using the Korean National Health Insurance Service (2010–2018) database. After matching by age and sex, 6129 SCI patients and 22,979 controls were included. The primary outcome was incident DM, and risk was evaluated for both the short term (within 1 year after SCI) and the long term (after 1 year of SCI diagnosis). Results: The risk of DM was higher among patients with cervical- and thoracic-level SCI accompanied by disability compared to the controls during follow-up (4.6 ± 2.6 years). The short-term risk of DM was higher among patients with SCI (odds ratio [OR] 2.51, 95% confidence interval [CI] 1.91–3.27) than among the controls and it was even higher among patients with severe disability (OR 5.38, 95% CI 2.91–9.27). According to the level of injury, patients with cervical SCI had the highest short-term risk of DM (with disability, OR 4.93, 95% CI 3.07–7.63). There was no significant increase of DM risk in the long term, after 1 year of SCI diagnosis. Conclusions: Patients with SCI accompanied by severe disability and cervical-level injury had higher risks of pronounced DM in the short term. The findings of this study emphasize the need for active surveillance of DM among patients with high-level SCI and disability, especially in the short term, in addition to continuous monitoring and proper management of DM in the long term.

## 1. Introduction

Spinal cord injury (SCI) is a morbid condition that can cause immobility or disability. According to the Global Burden of Disease (GBD), there were approximately 20.6 million patients with SCI worldwide in 2019 [1]. Patients with SCI can suffer from various comorbidities or secondary health conditions, including diabetes mellitus (DM) [2,3,4,5].

DM is a chronic disease that can compromise quality of life and cause multi-organ damage, resulting in increased morbidity [6]. Patients with SCI might be at risk of DM because they are commonly exposed to steroid administration during the acute phase of injury [7,8] and may have disabilities that cause immobility and decreased physical activity [1,9]. Furthermore, the occurrence of DM can be associated with the prognosis of patients with SCI; for example, wound infection, pneumonia, sepsis, or cardiovascular disease [3,10].

The prevalence of DM among patients with SCI ranges from 13.8% to 49.7% (Supplementary Table S1) [11,12,13,14,15,16,17,18,19,20]; however, most studies were based on cross-sectional designs [12,14,15] or were conducted among patients with chronic SCI [11,13,14,15,17,19,20] and had limited data on the level and extent of SCI [12,15,16]. Thus, it is uncertain whether patients with SCI are at a higher risk of DM either in the short term or in the long term after SCI diagnosis compared to the general population, depending on the level and extent of SCI.

According to a retrospective cohort study in Taiwan, patients with SCI had a higher risk of DM (adjusted hazard ratio [aHR] 1.33, 95% confidence interval [CI] 1.22–1.45) [21]. To our knowledge, that study is the only one to investigate the incidence of DM after SCI compared with a control group matched by age and sex. However, that study did not distinguish between the short-term and the long-term, in terms of association with risk of DM. Moreover, there was limited adjustment of confounders; important risk factors for DM including body mass index (BMI), smoking status, or alcohol consumption, which are reported to be associated with altered glucose metabolism [2,4,12] were not incorporated into the analyses.

In this regard, we aimed to investigate both the short-term and long-term risks of DM among patients with SCI in comparison to a matched control group with adjustment for traditional DM risk factors considering the presence or severity of post-SCI disability and the level of injury.

## 2. Materials and Methods

### 2.1. Data Source

This study was conducted based on the Korean National Health Insurance System (KNHIS) database from 2010–2018. The KNHIS is a non-profit health insurance system managing reimbursements of medical billing. Approximately 97% of Koreans are subscribers of the KNHIS, and employed individuals are required to pay insurance premiums assessed by their income. The remaining 3% of Koreans at the lowest income level are covered by the Medicaid program. Koreans who pay insurance premiums and individuals who are 40 years or older can be provided with biennial national general health screenings. Thus, the KNHIS database contains health information including sociodemographic data, anthropometric measurements, laboratory tests, disease diagnoses, prescription of drugs, and health behavior characteristics [22].

### 2.2. Study Design and Population

We initially included 18,239 patients who were newly diagnosed with cervical SCI (S14), thoracic SCI (S24), or lumbar SCI (S34) based on the International Classification of Disease (ICD-10) codes during their hospitalization between 1 January 2010 and 31 December 2018. Among patients with SCI who participated in national general health screenings within two years before SCI diagnosis (n = 8415), we excluded individuals who were younger than 20 years (n = 2), who were diagnosed with DM before SCI diagnosis (n = 1754), who had fasting glucose level more than 126 mg/dL (n = 219), and who had missing data (n = 311). Finally, a total of 6129 patients with SCI were included in this study.

**Figure 1:**
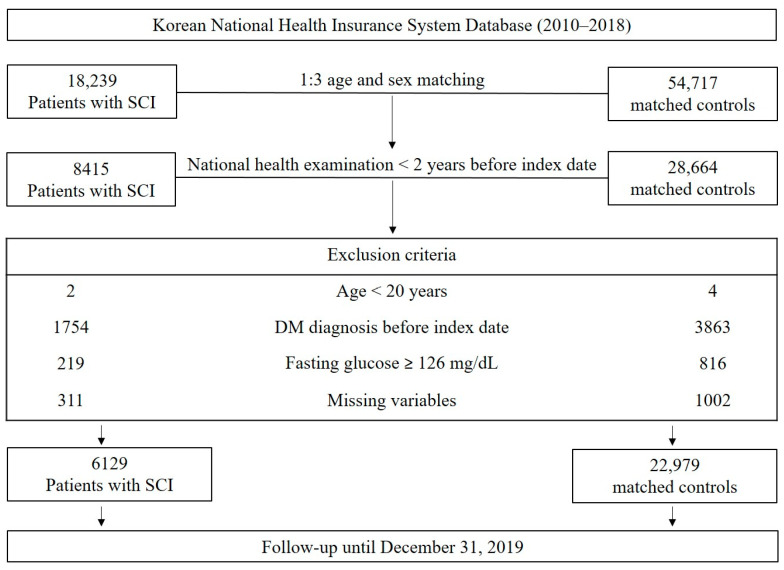
Flow chart of study population enrollment.

We conducted 1:3 age- and sex-matching to select the control group. We further applied the same exclusion criteria, which resulted in a total of 22,979 individuals comprising the control group (Figure 1). Each control participant was assigned the index date corresponding to the matched SCI patient’s date of SCI diagnosis.

This study was approved by the Institutional Review Board of Samsung Medical Center (IRB File No. SMC 2020-12-068). The requirement of written informed consent was waived as the data were de-identified under confidentiality guidelines.

### 2.3. Primary Outcome

The primary outcome was the incidence of DM, which was based on ICD-10 codes (E11–E14) and the prescription of antidiabetic medications. We followed study participants from the date of SCI diagnosis (or the index date) until the first incidence of DM, censoring, death, or 31 December 2019.

### 2.4. Status and Severity of Disability after SCI

We defined those who were registered with the Korea National Disability Registration System (KNDRS) within 1 year after SCI diagnosis as SCI patients with disability. Patients with disability persisting for 6 months or more can be registered in the KNDRS after evaluations by rehabilitation specialists, neurologists, or neurosurgeons to get social benefits from the Korean government. The KNDRS database thus contains records of the degree of functional losses or clinical impairment of patients with 15 types of disabilities, including physical and mental disabilities. Based on the extent of impairment in the affected extremities and the degree of paralysis according to the manual muscle strength test (MMT) provided by certified medical professionals, physical disability grades are determined from grade 1 (most severe) to 6 (least severe) (Supplementary Tables S2 and S3) [23]. The present study classified the severity of disability into two groups: severe (grades 1–3) and mild (grades 4–6), which was used in previous studies using the KNDRS database [24,25].

### 2.5. Covariates

Income level based on the monthly insurance premium was classified into two groups: the lowest quartile vs. the three higher quartiles. Place of residence was categorized into two groups: urban and rural. BMI was calculated by dividing body weight by the square of height (kg/m^2^). Hypertension (I10–I11) and dyslipidemia (E78) were defined based on claims data, according to ICD-10 codes prior to the screening date. Prescription records of antihypertensive or lipid-lowering medications were also considered, to identify hypertension or dyslipidemia. The Charlson Comorbidity Index (CCI) was identified based on ICD-10 codes to evaluate the overall comorbidity load [26]. Systolic blood pressure, diastolic blood pressure, and laboratory data after overnight fasting including fasting serum glucose, total cholesterol, and estimated glomerular filtration rate (eGFR) were measured from national general health screenings. Smoking status was divided into three groups: never-smokers, ex-smokers, and current smokers. We classified alcohol consumption into three groups: none, mild drinkers (<30 g/day), and heavy drinkers (≥30 g/day). Regular exercise was defined as vigorous activity for more than 20 min per session ≥3 days per week or moderate-intensity activity for more than 30 min per session ≥5 days per week. 

### 2.6. Statistical Analyses

We conducted descriptive statistics using Student’s *t*-tests for continuous variables, and chi-square tests for categorical variables, to investigate the sociodemographic, clinical, and health behavior characteristics of patients with SCI and those of the matched controls. We further performed analysis of variance (ANOVA) or chi-square tests to investigate the differences among patients with and without post-SCI disability and the controls.

For evaluation of the short-term risk of DM, we estimated ORs of DM within 1 year from SCI diagnosis or the index date among SCI patients and the controls. Analyses were performed according to the presence or severity of post-SCI disability and the level of injury.

For evaluation of the long-term risk of DM, we applied a 1-year lag period, excluding those who died or developed DM within 1 year after SCI diagnosis, to evaluate the risk of DM after SCI stabilization. Cox proportional hazards regression analysis was performed with adjustment for the confounders of age, sex, income, residence, BMI, hypertension, dyslipidemia, fasting serum glucose, smoking status, and alcohol consumption. Kaplan–Meier curves were plotted to illustrate the cumulative incidence of DM. 

SAS software version 9.4 (SAS Institute Inc., Cary, NC, USA) was used for all statistical analyses with the level of statistical significance set at <0.05.

## 3. Results

### 3.1. Baseline Characteristics

The mean follow-up durations were 4.6 ± 2.6 years and 5.0 ± 2.5 years for patients with SCI and the controls, respectively. Among patients with SCI, the mean age was 56.8 ± 13.2 years, and the proportion of males was 68.1%; this was comparable to the mean age of 56.6 ± 13.3 and the 69.3% proportion of males in the control group. Patients with SCI were more likely to be current smokers, heavy alcoholics, and in the lower income group. Higher mean CCI and higher prevalence of hypertension/dyslipidemia were found among patients with SCI compared to the controls. Among patients with SCI, those with disabilities (n = 1103) were older, had higher CCI, and higher prevalence of hypertension/dyslipidemia than those without disability (n = 5026) (Table 1).

### 3.2. Short-Term Risk of DM among Patients with SCI Compared to the Controls

The risk of DM within 1 year of SCI diagnosis or the index date was higher among patients with SCI (OR 2.51, 95% CI 1.91–3.27) than the controls. The risk of DM was different according to post-SCI disability status; patients with disability showed a higher risk of DM than the controls (OR 4.55, 95% CI 2.97–6.79), and the risk was even higher among patients with severe disability (OR 5.38, 95% CI 2.91–9.27). According to the level of injury, patients with cervical SCI showed a higher risk of DM than the controls (with disability, OR 4.93, 95% CI 3.07–7.63; without disability, OR 2.42, 95% CI 1.75–3.31), while patients with thoracic or lumbar SCI did not show a significantly increased risk of DM (Table 2).

### 3.3. Long-Term Risk of DM among Patients with SCI Compared to Controls

**Figure 2:**
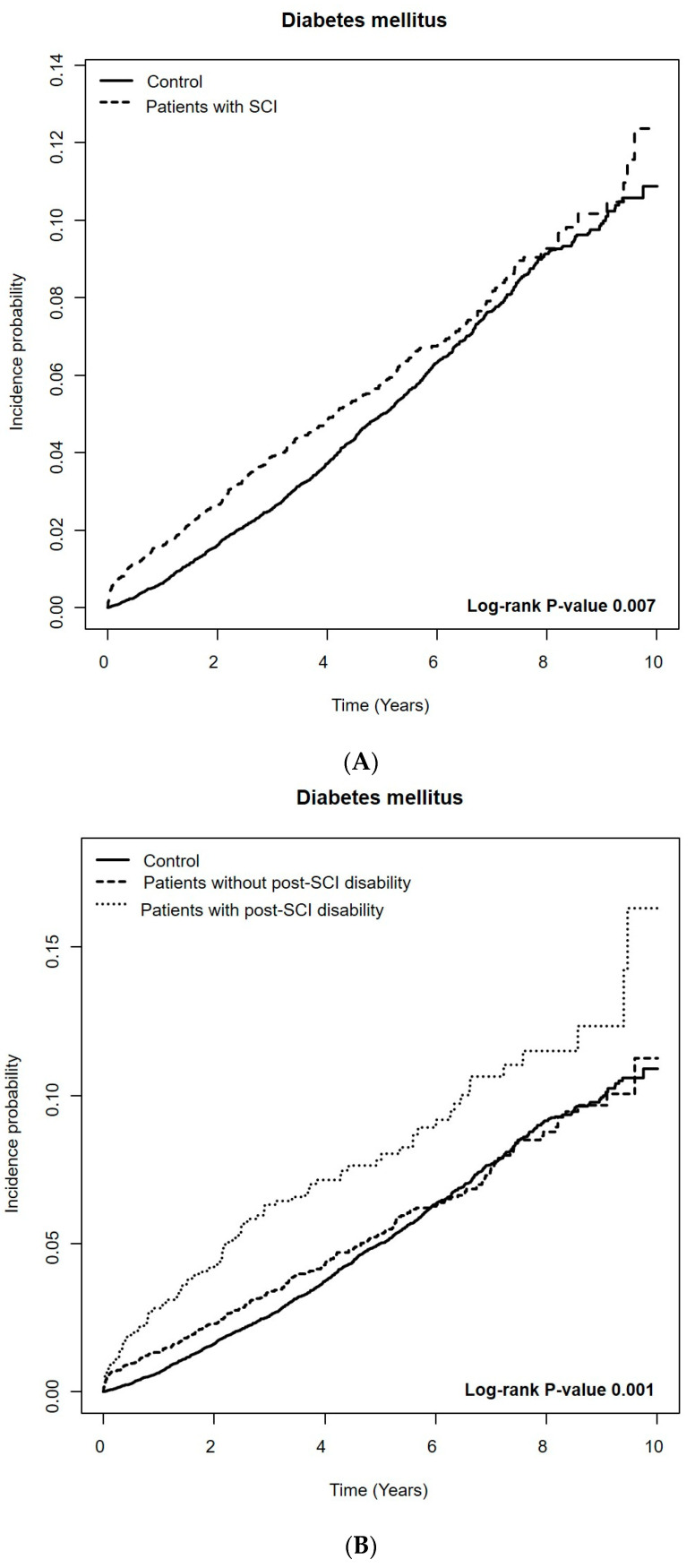
Kaplan–Meier curves of incidence probability of diabetes mellitus among patients with spinal cord injury (SCI) and the matched controls. (**A**) Patients with SCI compared to the matched controls. (**B**) Patients with and without post-SCI disability, compared to the matched controls.

The incidence probability of DM among patients with SCI compared to that of the controls is demonstrated in Figure 2. We found a higher risk of DM among patients with SCI than the controls during the total follow-up period (aHR 1.13, 95% CI 1.00–1.27). The risk of DM was further increased for those with post-SCI disability (aHR 1.41, 95% CI 1.14–1.74) and those with severe post-SCI disability (aHR 1.83, 95% CI 1.34–2.51). Patients with cervical or thoracic SCI accompanied by post-SCI disability showed an increased risk of DM (aHR 1.42, 95% CI 1.11–1.82 and aHR 1.92, 95% CI 1.06–3.47, respectively) (Table 3).

However, when we applied a 1-year lag period to exclude those who died or were diagnosed with DM within the first year of SCI diagnosis, we did not observe a significant increase of DM risk (aHR 0.93, 95% CI 0.86–1.06), even in the presence of post-SCI disability (aHR 1.05, 95% CI 0.81–1.37) and regardless of its severity. Patients with post-SCI disability and thoracic-level injury showed the highest risk of DM, but the result was not significant (aHR 1.65, 9% CI 0.82–3.31) (Table 3).

## 4. Discussion

### 4.1. Investigation into Risk of DM among Patients with SCI

We investigated both short-term and long-term risks of DM among patients with SCI according to the presence or severity of post-SCI disability and the level of injury. The short-term risk of DM was higher among patients with SCI than it was among the controls, and the risk was further increased when patients were affected by severe post-SCI disability or cervical SCI. We did not find a significant increase of DM risk among patients with SCI compared to the controls in the long term after applying a 1-year lag, regardless of the presence or the severity of disability.

The higher short-term risk of DM among patients with SCI may be attributed to the alteration of glycemic control mechanisms related to steroid administration after SCI. Steroid administration has been used to prevent complications of SCI considering its anti-inflammatory and neuroprotective effects, including improving ischemia, reducing spinal cord edema, inhibiting the release of inflammatory cytokines, and reducing the apoptosis of neurons during the acute phase of SCI [7,8,27]. During steroid administration, serum glucose or glycated hemoglobin levels sharply increased [28,29]. Despite disparities in clinical guidelines because of concerns regarding the increased risk of wound infection, sepsis, or pulmonary embolism caused by steroids [7,28,29], the prescription rate of high-dose steroids was 59% between 2007 and 2017, according to a Korean population-based study of acute SCI [7]. Thus, patients are likely to be exposed to a hyperglycemic state in the acute to subacute phase of SCI. Therefore, active surveillance and proper management of DM may benefit patients with SCI, who are likely to receive steroid treatment in the immediate period after SCI diagnosis.

Regarding the severity of disability and level of injury, we observed the highest DM risk among patients with SCI accompanied by severe disability and cervical level injury in the short term. A reduced phosphoinositide 3-kinase signaling pathway in the hypothalamus after SCI may be one of the plausible mechanisms of peripheral inflammation and insulin resistance [30]. According to previous studies, SCI can cause lower motor neuron injury resulting in flaccid paralysis and muscle atrophy [31,32], which leads to mitochondrial dysfunction, impaired glucose tolerance, insulin resistance [33,34,35], and systematic inflammation [36]. A somatic nerve injury induces skeletal muscle atrophy as early as 6 weeks after SCI [37]. Furthermore, a visceral nerve injury also can be associated with disruption of hormone secretion including testosterone, growth hormone and insulin-like growth factor (IGF-1), which aggravate skeletal muscle atrophy [33,38]. In a previous study, it was reported that exogenous testosterone treatment may promote the recovery of skeletal muscle atrophy among male patients with low levels of testosterone after SCI [32,39]. Growth hormone may be associated with muscle regeneration, by inducing production of IGF-1, which regulates muscle metabolism. Considering SCI above T6 usually accompanies a broad extent of neurologic dysfunction or immobility [27,40], patients with high-level injury and severe disability are more likely to be at risk of physical inactivity, which aggravates the alteration of glucose metabolism and loss of endocrine function [10,41,42]. Moreover, impaired mobility and decreased physical activity can cause body composition changes, including a loss of lean muscle, accumulation of adipose tissue, and increase in body fat percentage, which can lead to neurogenic obesity associated with insulin resistance or DM [2,3,4,20,27,43].

According to a cohort study in Taiwan, patients with SCI had a higher overall risk of DM than control participants, but analysis for the risk of DM began at the date of SCI diagnosis and did not distinguish the risk in terms of short-term or long-term [21]. In contrast, we applied a 1-year lag period to estimate the long-term risk of DM, to exclude a possible surveillance bias during the immediate period post-SCI diagnosis. In this study, we did not find a significant increase of DM risk in the long term. However, we should not underestimate the importance of glycemic control beyond the short-term period after SCI. Chronic inflammation and neurogenic obesity might be associated with metabolic or cardiovascular disease risk among patients with SCI [10,12] in the course of SCI. Patients with chronic SCI are susceptible to neurogenic obesity [4,44,45]. Moreover, various interventions including pharmacotherapy, exercise, diet therapy, neuromuscular electric stimulation, and bariatric surgery were conducted to reduce body weight and manage obesity [45]. Patients in the chronic phase of SCI are less likely to be treated with a high dose of steroids compared to patients in the acute or subacute phase of SCI, especially if the etiology is traumatic [7,46]. Considering that we defined the diagnosis of SCI based on ICD-10 codes S14, S24, and S34, which usually correspond to traumatic SCI [7,47], the chronic SCI patients included in this study might receive less frequent steroid administration than non-traumatic SCI patients, after the acute or subacute phase [12,46]. Furthermore, patients with risk factors of DM who are susceptible to hyperglycemia or insulin resistance might be diagnosed with DM earlier during hospitalization and acute care following SCI diagnosis, making the risk less apparent after applying a 1-year lag period. Moreover, we cannot ignore proper monitoring and management of DM by clinicians during the patient’s regular presentation at hospitals or clinics.

Regarding the association of the level of injury with long-term risk of DM, we found a higher risk among patients with thoracic SCI compared with the controls, although the difference was not significant. However, considering the small number of patients with thoracic SCI (n = 8 with disability; n = 12 without disability) in this study, the result might be related with insufficient statistical power. According to a previous study, a high-level SCI can result in severe disability associated with loss of descending control of spinal circuits below the injury level [10,12]. In tetraplegic patients, a high level of leptin and a high risk of neurogenic obesity were associated with complete disruption of the sympathetic nervous system [3,48].

### 4.2. Strengths

To the best of our knowledge, this is the first nationwide population-based study to investigate the short-term and long-term risks of DM among patients with SCI compared with a matched control group considering disability status and level of injury. No previous studies assessed both short-term and long-term risk of DM among patients with SCI. We observed a higher risk of DM especially in the short term. Furthermore, we conducted statistical analyses adjusting for traditional risk factors for DM, including sociodemographic factors, comorbidities, and health behaviors that can affect glucose metabolism and the incidence of DM.

### 4.3. Limitations

Nevertheless, there are several limitations to be addressed. First, this study is based on the KNHIS database, which is a nationwide systematic database of Koreans not exclusive to patients with SCI. Despite the information on the American Spinal Injury Association Impairment Scale, a detailed extent of SCI could not be collected. However, we used the KNDRS database to identify the presence and severity of disability, which were assessed by certified medical professionals. Second, we could not reflect changes of risk factors for DM during follow-up. The baseline characteristics of the study participants were based on the national general health screening examinations performed within 2 years before the index date. Further studies are warranted, to consider the evolution of risk factors in the course of SCI. Third, we could not directly assess the effects of steroid treatment on glucose metabolism because we could not gather information on steroid administration including the cumulative dose of steroid, and serial serum glucose levels. Fourth, since we defined the incidence of DM based on claims data based on ICD-10 codes and prescription data, the risk of DM in the long term may be underestimated. Fifth, our findings may not be generalizable to populations of other ethnicities.

## 5. Conclusions

We investigated both the short-term and long-term risks of DM among patients with SCI compared with a matched control group. In this study, patients with severe disability and cervical SCI had a higher risk of DM in the short term. The findings of this study emphasize the need for active surveillance of DM among patients with high-level SCI and disability, especially in the short term, in addition to continuous monitoring and proper management of DM in the long term.

## Figures and Tables

**Table 1:** Demographics of patients with spinal cord injury (SCI) and the matched controls.

	SCI			Control (n = 22,979)	*p*-Value ^a^	*p*-Value ^b^
	Total (n = 6129)	Without Disability (n = 5026)	With Disability (n = 1103)			
Age, years	56.8 ± 13.2	56.3 ± 13.3	59.0 ± 12.5	56.6 ± 13.3	0.271	<0.001
Sex, male, n (%)	4172 (68.1)	3379 (67.2)	793 (71.9)	15,928 (69.3)	0.061	0.002
Income, lowest 25%, n (%)	1279 (20.9)	1014 (20.2)	265 (24.0)	4042 (17.6)	<0.001	<0.001
Residence, urban, n (%)	2288 (37.3)	1873 (37.3)	415 (37.6)	10,404 (45.3)	<0.001	<0.001
BMI, kg/m^2^	24.0 ± 3.2	24.0 ± 3.1	24.0 ± 3.4	24.0 ± 3.1	0.747	0.909
Hypertension, n (%)	2498 (40.8)	1969 (39.2)	529 (48.0)	8128 (35.4)	<0.001	<0.001
Dyslipidemia, n (%)	1734 (28.3)	1402 (27.9)	332 (30.1)	6071 (26.4)	0.003	0.004
Charlson Comorbidity Index	2.7 ± 2.3	2.5 ± 2.2	3.5 ± 2.3	1.2 ± 1.5	<0.001	<0.001
Systolic blood pressure (mmHg)	124.7 ± 15.3	124.6 ± 15.1	125.2 ± 15.9	124.3 ± 14.6	0.080	0.103
Diastolic blood pressure (mmHg)	77.3 ± 10.0	77.2 ± 10.0	77.6 ± 10.2	77.0 ± 9.76	0.103	0.142
Fasting serum glucose (mg/dL)	95.5 ± 11.5	95.4 ± 11.3	96.0 ± 12.0	95.3 ± 11.3	0.207	0.135
Total cholesterol (mg/dL)	194.8 ± 37.6	195.6 ± 37.4	191.3 ± 38.0	197.2 ± 36.5	<0.001	<0.001
eGFR (mL/min/1.73 m^2^)	92.1 ± 49.3	91.8 ± 50.1	93.7 ± 45.3	89.0 ± 44.3	<0.001	<0.001
Regular exercise, n (%)	1290 (21.1)	1056 (21.0)	234 (21.2)	5057 (22.0)	0.106	0.268
Smoking, n (%)					<0.001	<0.001
Never-smoker	3069 (50.1)	2522 (50.2)	547 (49.6)	11,906 (51.8)		
Ex-smoker	1182 (19.3)	942 (18.7)	240 (21.8)	5388 (23.5)		
Current smoker	1878 (30.6)	1562 (31.1)	316 (28.7)	5685 (24.7)		
Alcohol Consumption, n (%)					<0.001	<0.001
None	2947 (48.1)	2359 (46.9)	588 (53.3)	11,681 (50.8)		
Mild (<30g/d)	2404 (39.2)	2007 (39.9)	397 (36.0)	9363 (40.8)		
Heavy (≥30g/d)	778 (12.7)	660 (13.1)	118 (10.7)	1935 (8.4)		
Follow-up duration, years	4.6 ± 2.6	4.6 ± 2.6	4.7 ± 2.6	5.0 ± 2.5	<0.001	<0.001

SCI, spinal cord injury; BMI, body mass index. Data are presented as mean ± standard deviation (SD) or number (%). ^a^ Comparison between patients with SCI and the controls using Student’s *t*-tests or chi-square tests. ^b^ Comparison among patients without post-SCI disability, patients with post-SCI disability, and the controls, using analysis of variance (ANOVA) or chi-square tests.

**Table 2:** Incidence rates (IR) ^a^ and odds ratios (OR) ^b^ of diabetes mellitus among patients with spinal cord injury (SCI) compared to the matched controls in the short term.

	Events	IR	OR (95% Confidence Interval)
Comparison between patients with SCI and the controls			
Control	145	0.006	1.00 (ref.)
SCI	96	0.016	2.51 (1.91, 3.27)
Comparison between patients with SCI and the controls by disability status			
Control	145	0.006	1.00 (ref.)
SCI without disability	65	0.013	2.06 (1.51, 2.79)
SCI with disability	31	0.028	4.55 (2.97, 6.79)
Comparison between patients with SCI and the controls by the degree of disability			
Control	145	0.006	1.00 (ref.)
SCI without disability	65	0.013	2.06 (1.51, 2.79)
SCI with mild disability (Grade 4–6)	16	0.025	3.98 (2.20, 6.73)
SCI with severe disability (Grade 1–3)	15	0.033	5.38 (2.91, 9.27)
Comparison between patients with SCI and the controls by the level of SCI			
Control	145	0.006	1.00 (ref.)
SCI without disability			
Cervical level	58	0.015	2.42 (1.75, 3.31)
Thoracic level	3	0.012	1.98 (0.40, 5.99)
Lumbar level	4	0.004	0.67 (0.18, 1.75)
SCI with disability			
Cervical level	25	0.030	4.93 (3.07, 7.63)
Thoracic level	3	0.023	3.78 (0.76, 11.52)
Lumbar level	3	0.020	3.17 (0.64, 9.63)

SCI, spinal cord injury. ^a^ Incidence rates per 1000 person–years ^b^ Odds ratios.

**Table 3:** Incidence rates (IR) ^a^ and adjusted hazard ratios (aHR) ^b^ of diabetes mellitus among patients with spinal cord injury (SCI) compared to the matched controls.

	Total Follow-Up Period			After a 1-Year Lag Period		
	Events	IR	aHR (95% CI)	Events	IR	aHR (95% CI)
Comparison between patients with SCI and the controls						
Control	1227	10.7	1.00 (ref.)	1082	11.8	1.00 (ref.)
SCI	354	12.5	1.13 (1.00, 1.27)	258	11.5	0.93 (0.81, 1.06)
Comparison between patients with SCI and the controls by disability status						
Control	1227	10.7	1.00 (ref.)	1082	11.8	1.00 (ref.)
SCI without disability	264	11.4	1.05 (0.92, 1.20)	199	10.9	0.93 (0.81, 1.06)
SCI with disability	90	17.4	1.41 (1.14, 1.74)	59	14.4	1.05 (0.81, 1.37)
Comparison between patients with SCI and the controls by the degree of disability						
Control	1227	10.7	1.00 (ref.)	1082	11.8	1.00 (ref.)
SCI without disability	264	11.4	1.05 (0.92, 1.20)	199	10.9	0.93 (0.81, 1.06)
SCI with mild disability (Grade 4–6)	50	16.2	1.19 (0.89, 1.58)	34	13.9	0.91 (0.65, 1.29)
SCI with severe disability (Grade 1–3)	40	19.0	1.83 (1.34, 2.51)	25	15.0	1.32 (0.89, 1.96)
Comparison between patients with SCI and the controls by the level of SCI						
Control	1227	10.7	1.00 (ref.)	1082	11.8	1.00 (ref.)
SCI without disability						
Cervical level	201	11.7	1.06 (0.91, 1.23)	143	10.6	0.86 (0.72, 1.02)
Thoracic level	15	15.0	1.17 (0.70, 1.95)	12	15.5	1.10 (0.62, 1.94)
Lumbar level	48	9.60	0.98 (0.74, 1.32)	44	10.8	0.98 (0.73, 1.33)
SCI with disability						
Cervical level	68	18.0	1.42 (1.11, 1.82)	43	14.5	1.03 (0.76, 1.39)
Thoracic level	11	18.8	1.92 (1.06, 3.47)	8	17.4	1.65 (0.82, 3.31)
Lumbar level	11	13.3	1.05 (0.58, 1.91)	8	11.9	0.85 (0.42, 1.70)

SCI, spinal cord injury. ^a^ Incidence rates per 1000 person-years. ^b^ Adjusted for age, sex, income, residence, body mass index, hypertension, dyslipidemia, fasting serum glucose, smoking status and alcohol consumption.

## Data Availability

The data reported in this study are not available to the public. They are available only to licensed researchers authorized by the KNHIS.

## Supplementary Materials

The following supporting information can be downloaded at: https://www.mdpi.com/article/10.3390/healthcare12181859/s1, Table S1. Summaries of previous studies on the association between spinal cord injury (SCI) and diabetes mellitus (DM). Table S2. Definitions of the degree of severity in dysfunctions of the upper extremities. Table S3. Definitions of the degree of severity in dysfunctions of the lower extremities.

## Abbreviations

DM	diabetes mellitus
SCI	spinal cord injury
KNHIS	Korean National Health Insurance System
KNDRS	Korea National Disability Registration System
ICD-10	International Classification of Diseases, 10th revision
eGFR	estimated glomerular filtration rate
IGF-1	insulin-like growth factor
CCI	Charlson comorbidity index
OR	odds ratio
aHR	adjusted hazard ratio
CI	confidence interval
