# Supplementary material for: Short-Term and Long-Term Risk of Diabetes Mellitus among Patients with Spinal Cord Injury: A Nationwide Retrospective Cohort Study
Source: Healthcare (Basel). 2024 Sep 15;12(18):1859. doi: 10.3390/healthcare12181859 (PMC11431731; doi:10.3390/healthcare12181859)
Supplement: Supplementary file 1 [file healthcare-12-01859-s001.zip › healthcare-3169928-supplementary.pdf]

Supplementary Table S1. Summaries of previous studies on the association between spinal cord injury (SCI) and diabetes mellitus (DM)

Author (et al.)	Year	Country/ Cohort	Subjects with SCI (N)	Subjects without SCI (N)	Mean age (years±SD)	SCI type	Mean follow-up (years)	Outcome assessment	Adjustment for covariates	Outcome	Significance
**Cross-sectional study**											
Jörgensen	2019	Sweden<br><br>Swedish Aging with Spinal Cord Injury Study (SASCIS)	123 (total)<br>87 (male)<br>36 (female)	-	63±9	Duration:<br>Chronic,<br>24±12 years<br>Etiology:<br>Not specified<br>Level:<br>C1-L5<br>Extent:<br>AIS A-D	-	Interview, medical record	-	**T2DM prevalence**<br>16%: previous history of DM<br>15%: fasting glucose ≥ 7 mmol/L	The high occurrence of cardiovascular risk factors among older adults with long-term SCI.<br><br>Regular assessments and interventions targeting cardiovascular risk are warranted.
Cragg	2013	Canada<br><br>Canadian Community Health Survey (CCHS) 2010 Annual Component.	353 (total)	60,325	40–44 (median)	Duration:<br>Not specified<br>Etiology:<br>Not specified<br>Level:<br>Not specified<br>Extent:<br>Not specified	-	Interview	aOR1<br>age, sex<br><br>aOR2<br>aOR1+ sex, BMI<br><br>aOR3<br>aOR2+ HTN, smoking, alcohol intake, daily physical activity, daily fruits/vegetables intake	**T2DM prevalence**<br>● Non-SCI 5.91%<br>● SCI 13.66%<br><br>**T2DM ORs**<br>● Non-SCI<br>aOR 1.00 (ref.)<br>● SCI<br>unadjusted OR<br>2.52<br>(95%CI: 1.81, 3.52)<br>aOR1<br>1.66<br>95%CI: 1.16, 2.36)<br>aOR2<br>1.65<br>95%CI: 1.13, 2.42)<br>aOR3<br>2.45<br>(95%CI: 1.34, 4.47)	There is a strong association between SCI and T2DM.
LaVela	2012	USA<br><br>Paralyzed Veterans of America membership/CDC BRFSS database	794 (male)	Non-SCI Veteran 13,528 (men)<br><br>General population 6,105 (men)	SCI 74<br><br>Non SCI veteran 74<br><br>Non SCI general population 73	Duration: Chronic (≥20yr)<br>Etiology:<br>Not specified<br>Level: Tetraplegia Paraplegia<br>Extent:<br>Not specified	-	Self-reported assessment (SCD-HCQ, BRFSS)	Age, race, education, marital status, smoking, drinking, high blood pressure, high cholesterol level, group status (SCI vs non-SCI veteran, SCI vs general population)	**DM prevalence**<br>● General population 17.86%<br>● Non-SCI veteran 19.26%<br>● SCI 20.30%<br><br>**DM aORs**<br>● Non-SCI veteran<br>aOR 1.00 (ref.)<br>● SCI<br>aOR 0.98 (95%CI: 0.80, 1.20)<br><br>● General population<br>aOR 1.00 (ref.)<br>● SCI aOR 1.16<br>(95%CI: 0.93, 1.45)	Prevalence of DM among older adult men with SCI is similar to that of other older adult men.

**Case-control study**											
Selassie A.	2013	USA<br><br>62 acute care nonfederal hospitals in South Carolina<br>* Multicenter	1,776<br>(total)<br>1,408<br>(male)<br>368<br>(female)	1,780<br>(LEF total)<br>1,098<br>(LEF male)<br>682<br>(LEF female)	SCI<br>38.8±15.0<br><br>LEF<br>35.1±16.4	Duration:<br>Acute<br>Etiology:<br>Traumatic<br>Level:<br>Not specified<br>Extent:<br>ISS (1-75)	12	ICD-9-CM	ISS	**DM aOR**<br>OR 1.68<br>(95%CI: 1.01, 2.79)	Patients with traumatic SCI have significantly higher odds of DM, heart disease, hypertension, and obesity than persons with LEF at the onset of SCI.
**Prospective cohort study**											
Solinsky R.	2022	USA<br><br>5 National Institute on Disability, Independent Living, and Rehabilitation Research Model SCI Rehabilitation Centers<br>* Multicenter	98<br>(total)<br>78<br>(male)<br>17<br>(female)	1,609<br>(total)<br>1,311<br>(male)<br>298<br>(female)	SCI<br>37.3±13.9<br><br>Control<br>37.8±14.4	Duration:<br>Acute<br>(2.7±1.1mo)<br>Etiology:<br>Traumatic<br>Level:<br>C2-L2<br>(C2-8, T1-6, T7-L2)<br>Extent:<br>AIS A-D	-	Medical record<br><br>Fasting serum (mg/dL)<br><br>Insulin (uIU/mL)<br><br>HbA1C (%)	-	**Insulin resistance prevalence**<br>● Control<br>42.0%<br>● SCI<br>37.5%<br>- Elevated fasting glucose:<br>12.5%<br>- HOMA(v.2) criteria:<br>33.3%	Insulin resistance was not significantly different between patients with SCI and the controls.
**Retrospective cohort study**											
Cao Y.	2020	USA<br><br>South Carolina SCI Surveillance Registry (SCSCISR)<br>(2010-2013)	501<br>(total)<br>70%<br>(male)<br>30%<br>(female)	-	41.84±17.41<br>(at injury year)	Duration:<br>Chronic (≥1yr)<br>Etiology:<br>Traumatic<br>Level:<br>C1-C4<br>C5-C8<br>Non-cervical<br>Extent:<br>Not specified	4.3±1.0	Self-reported assessment (BRFSS)	-	**DM prevalence**<br>14% (baseline)<br>17% (follow-up)	Chronic health conditions including DM were common among adults with traumatic SCI and increased significantly over time.
DiPiro N.D.	2019	USA<br><br>South Carolina SCI Surveillance Registry (SCSCISR)	787<br>(total)<br>568<br>(male)<br>219<br>(female)	-	49.7±16.7	Duration:<br>Chronic (≥1yr)<br>Etiology:<br>Traumatic<br>Level/Extent:<br>C1–C4, non-ambulatory<br>C5–C8, non-ambulatory<br>Non-cervical, non-ambulatory<br>Ambulatory (any	Not specified	Self-reported assessment (BRFSS)<br><br>Administrative billing data	-	**DM prevalence**<br>15.8%	Chronic health conditions including DM were common among adults with traumatic SCI.

						level, able to walk<br>< 1000 feet)<br>Ambulatory<br>(any level, able to<br>walk >1000 feet)					
Peterson	2021	USA<br><br>Private insurance claims database, Clinformatics DataMart Database (OptumInsight, Eden Prairie, MN).	9,081<br>(total)<br>3,829<br>(male)<br>5,252<br>(female)	1,474,232<br>(total)<br>699,950<br>(male)<br>774,282<br>(female)	SCI<br>18–44yr:<br>15.2%<br>45–64 yr:<br>28.0%<br>≥65 yr:<br>56.7%<br><br>Control<br>18–44yr:<br>34.8%<br>45–64 yr:<br>34.8%<br>≥65 yr:<br>28.5	Duration:<br>Not specified<br>Etiology:<br>Traumatic<br>Level/Extent:<br>Not specified	SCI<br>6.0±1.7<br><br>Control<br>5.3±1.5	ICD-9-CM	Model 1:<br>Unadjusted<br>Model 2: age, sex, race, geographic region<br>Model 3: Model 2+Modified Elixhauser Comorbidity Index<br>Model 4: Model 3+education, income	**DM incidence**<br>● Control<br>9.2%<br>HR 1.00 (ref.)<br>● SCI<br>15.9%<br>Model 1: HR 1.72<br>(95%CI: 1.62-1.83)<br>Model 2: HR 1.16<br>(95%CI: 1.09-1.24)<br>Model 3: HR 0.99<br>(95%CI: 0.94-1.06)<br>Model 4: HR 1.00<br>(95%CI: 0.94-1.06)	Adults with SCIs have a significantly higher incidence of and risk for common cardiometabolic morbidities, as compared to adults without SCIs.
Lai Y.J.	2014	Taiwan<br><br>Taiwan's National Health Insurance Research Database (1997-2010)	52,420<br>(total)<br>33,312<br>(male)<br>19,108<br>(female)	209,680<br>(total)<br>133,248<br>(male)<br>76,432<br>(female)	SCI<br>51.7±18.3<br><br>Control<br>51.6±18.3	Duration:<br>Not specified<br>Etiology:<br>Not specified<br>Level/Extent<br>Cervical,<br>Complete thoracic,<br>Incomplete thoracic,<br>Lumbar/sacral/ coccygeal (L-S-Co)	SCI<br>5.98<br><br>Control<br>6.50	ICD-9-CM<br>(codes 250)	Age, sex, comorbidity	**DM incidence**<br>● Control<br>HR 1.00 (ref.)<br>● SCI<br>HR 1.33<br>(95%CI: 1.22-1.45)<br><br>**DM incidence by level of injury**<br>● Control<br>HR 1.00 (ref.)<br>● Cervical<br>HR 1.20<br>(95%CI: 1.06, 1.36)<br>● Complete thoracic<br>HR 2.35<br>(95%CI: 1.05, 5.23)<br>● Incomplete thoracic<br>HR 1.60<br>(95%CI: 1.34, 1.92)<br>● L-S-Co<br>HR 1.38<br>(95%CI: 1.21, 1.57)	Patients with SCIs possess higher risk of DM than non-SCI patients.
**Systematic review/Meta-analysis**											
Raguindin P.F.	2021	MEDLINE<br>EMBASE<br>Web of Science<br>Cochrane from inception until July 4, 2020	47 studies<br>(N=3,878)<br><br>Serum glucose<br>13 studies<br>(N=985)<br><br>Insulin	-	39.3 (median)	Duration:<br>Chronic<br>Etiology:<br>Not specified<br>Level:<br>-Tetraplegia/ paraplegia<br>- High/low paraplegia	-	Fasting serum glucose (mg/dL)<br><br>Insulin (uIU/mL)<br><br>HOMA-IR	Level of Injury	**Mean glucose levels**<br>● Tetraplegia:<br>95.9±19.7<br>● Paraplegia:<br>93.4±18.8<br>(WMD: −0.5, 95%CI: −1.9,1.0, I^2 37.5%)<br><br>**Mean serum insulin**	No differences in glucose metabolism markers was found.

			6 studies<br>(N=403)<br><br>HOMA-IR<br>4 studies<br>(N=214)			Extent: Complete<br>Incomplete				**levels**<br>● Tetraplegia:<br>9.8±5.4<br>● Paraplegia:<br>8.9±4.2<br>(WMD: −0.3, 95%CI: -1.8, 1.1, I^2 81.6%)<br><br>Pooled differences between the groups was not statistically significant	
Gorden	2021	USA	30 studies<br>(total)	12 studies	-	Duration:<br>Different by studies<br>Etiology:<br>Different by studies<br>Level:<br>Different by studies<br>Extent:<br>Different by studies	-	Literature review	-	**DM prevalence**<br>Different by studies	Reviewed current literature on the prevalence and likely mechanisms driving insulin resistance and T2DM among patients with SCI.<br><br>Explored the various assessments and diagnostic criteria used for insulin resistance and T2DM and discussed the effects of exercise and/or diet to mitigate disorders of carbohydrate metabolism brought on by neurogenic obesity.

SCI, spinal cord injury; AIS, ASIA Impairment Scale; ICD-9-CM, International Classification of Disease, 9th Revision, Clinical Modification; CDC BRFSS, Centers for Disease Control and Prevention Behavioral Risk Factor Surveillance System; SCD-HCQ, Spinal Cord Dysfunction Health Care; LEF, lower extremity fracture; ISS, injury severity score; HOMA-IR, homeostatic model assessment for insulin resistance; DM, diabetes mellitus; T2DM, type 2 diabetes mellitus; HTN, hypertension; BMI, body mass index; CCI, Charlson comorbidity index; SD, standard deviation; CI, confidence interval; OR, odd ratio; HR, hazard ratio; WMD, weighted mean difference.

Supplementary Table S2. Definitions of the degree of severity in dysfunctions of the upper extremities

Grade		Definitions
Level	Number	
1	1	Complete paralysis of both arms (manual muscle strength grade* 0, 1)
2	1	Complete paralysis of one arm (grade 0, 1)
	2	Minimal movement with both nearly paralyzed arms (grade 2)
	3	Complete paralysis of all fingers of both hands (grade 0, 1)
3	1	Paralysis of both arms, which are moderately mobile but not functional (grade 3)
	2	Complete paralysis of the thumb and 2nd finger of both hands (grade 0, 1)
	3	Complete paralysis of all fingers of one hand (grade 0, 1)
	4	Minimal movement with one paralyzed arm (grade 2)
4	1	Complete paralysis of the thumbs of both hands (grade 0, 1)
	2	Complete paralysis of the thumb and 2nd finger of one hand (grade 0, 1)
	3	Complete paralysis of three fingers of one hand, including the thumb or 2nd finger (grade 0, 1)
	4	Paralysis of four fingers of one hand including the thumb or 2nd finger, which are moderately mobile but not functional (grade 3)
5	1	Paralysis of one arm, which is moderately mobile but not functional (grade 3)
	2	Paralysis of the thumbs of both hands, which are moderately mobile but not functional (grade 3)
	3	Complete paralysis of the thumb of one hand (grade 0, 1)
	4	Paralysis of the thumb and 2nd finger of one hand, which are moderately mobile but not functional (grade 3)
	5	Paralysis of three fingers of one hand including the thumb or 2nd finger, which are moderately mobile but not functional (grade 3)
6	1	Paralysis of the thumb of one hand, which is moderately mobile but not functional (grade 3)
	2	Complete paralysis of two fingers of one hand, including the 2nd finger (grade 0, 1)
	3	Paralysis of two fingers of one hand including the thumb, which are moderately mobile but not functional (grade 3)
	4	Complete paralysis of the 3rd, 4th, and 5th fingers of one hand (grade 0, 1)

*Manual muscle strength grade: 5 (normal), complete range of motion against gravity with full resistance; 4 (good), complete range of motion against gravity with some resistance; 3 (fair), complete range of motion against gravity with no resistance; 2 (poor), complete range of motion with gravity eliminated; 1 (trace), evidence of slight contractility with no evidence of joint motion even with gravity eliminated; 0 (zero), no evidence of muscle contractility

Source: Kim M, Jung W, Kim SY, Park JH, Shin DW. The Korea National Disability Registration System. Epidemiol Health. May 11 2023:e2023053. doi:10.4178/epih.e2023053

Supplementary Table 3. Definitions of the degree of severity in dysfunctions of the lower extremities

Grade		Definitions
Level	Number	
1	2	Complete paralysis of both legs (manual muscle strength grade* 0, 1)
2	4	Minimal movement with both nearly paralyzed legs (grade 2)
3	5	Complete paralysis of one leg (grade 0, 1)
4	1	Paralysis of both legs, which are moderately mobile but not functional (grade 3)
	5	Slight movement with one nearly paralyzed leg (grade 2)
5	6	Paralysis of one leg, which is moderately mobile but not functional (grade 3)
5	7	Complete paralysis of all toes of both feet (grade 0, 1)

*Manual muscle strength grade: 5 (normal), complete range of motion against gravity with full resistance; 4 (good), complete range of motion against gravity with some resistance; 3 (fair), complete range of motion against gravity with no resistance; 2 (poor), complete range of motion with gravity eliminated; 1 (trace), evidence of slight contractility with no evidence of joint motion even with gravity eliminated; 0 (zero), no evidence of muscle contractility

Source: Kim M, Jung W, Kim SY, Park JH, Shin DW. The Korea National Disability Registration System. Epidemiol Health. May 11 2023:e2023053. doi:10.4178/epih.e20230
